# Neoadjuvant chemotherapy remodels the tumor immune microenvironment by increasing activated and cytotoxic T cell, decreasing B cells and macrophages in small cell lung cancer

**DOI:** 10.1186/s12967-023-04526-4

**Published:** 2023-09-21

**Authors:** Xiaoling Shang, Chenyue Zhang, Kai Wang, Haiyong Wang

**Affiliations:** 1 Cheeloo College of Medicine, Shandong University, Jinan, China; 2 Shandong Cancer Hospital and Institute, Shandong First Medical University and Shandong Academy of Medical Sciences, Jinan, China; 3 Department of Integrated Therapy, Fudan University Shanghai Cancer Center, Shanghai Medical College, Shanghai, China; 4 Department of Oncology, Shanghai Medical College, Fudan University, Shanghai, 200032 China; 5 Key Laboratory of Epigenetics and Oncology, Research Center for Preclinical Medicine, Southwest Medical University, Luzhou, China; 6 Department of Internal Medicine-Oncology, Shandong Cancer Hospital and Institute, Shandong First Medical University, Shandong Academy of Medical Sciences, Number 440, Ji Yan Road, Jinan, 250117 China

The incorporation of chemotherapy into the neoadjuvant setting has confers benefit in non–small-cell lung cancer (NSCLC) [1]. Furthermore, the combination of chemotherapy with immunotherapy has achieved pathological responses, which might be correlated with prolonged survival in NSCLC [2]. Despite accomplishments of neoadjuvant chemo-immunotherapy in the treatment of NSCLC, whether patients with SCLC can take advantage of this treatment regimen remains to be elucidated. Additionally, there is a lack of detailed characterization of effects of neoadjuvant chemotherapy (NAC) on the tumor microenvironment (TME) in SCLC, especially in the clinical pre-treatment and post-treatment setting. Therefore, in the present study, we explored the alterations in TME after NAC in SCLC by utilizing single-cell RNA sequencing analyses. Furthermore, we validated this by dissecting TME in a total of 8 patients with SCLC before and after treatment with EC/EP using biopsies and blood samples.

Single-cell RNA sequencing of samples from a total of 11 SCLC patients (2 underwent chemotherapy before surgical resection and 9 did not received chemotherapeutic treatment before surgical resection) were analyzed [3]. Quality control of single-cell RNA sequencing were conducted (Additional file 1: Fig. S1A–C). Detailed methods were demonstrated in the Additional file 7: Methods. Different SCLC cases were shown to have different proportions of immune cells and cell clusters were identified and then labeled using tSNE plot (Additional file 1: Fig. S1D). The associated cell type was speculated via the transcript features and the corresponding markers have been employed to annotate cell type, as shown in Fig. 1A. Comparisons of subtypes of cells within the TME were demonstrated between SCLC patients subject to NAC and those without NAC treatment, which revealed that T cell proliferative were enriched in patients with SCLC without NAC **(**Fig. 1B**)**. As shown in Fig. 1C, more infiltration of activated and cytotoxic CD8+T cells and less immersion of T cell proliferative, plasma B cells and macrophages were observed in NAC-treated patients with SCLC. Further analysis of the major immune cell composition within TME in each patient demonstrated that patients with SCLC undergoing NAC have more activated and cytotoxic CD8+T cells (Additional file 1: Fig. S1E).

**Fig. 1:**
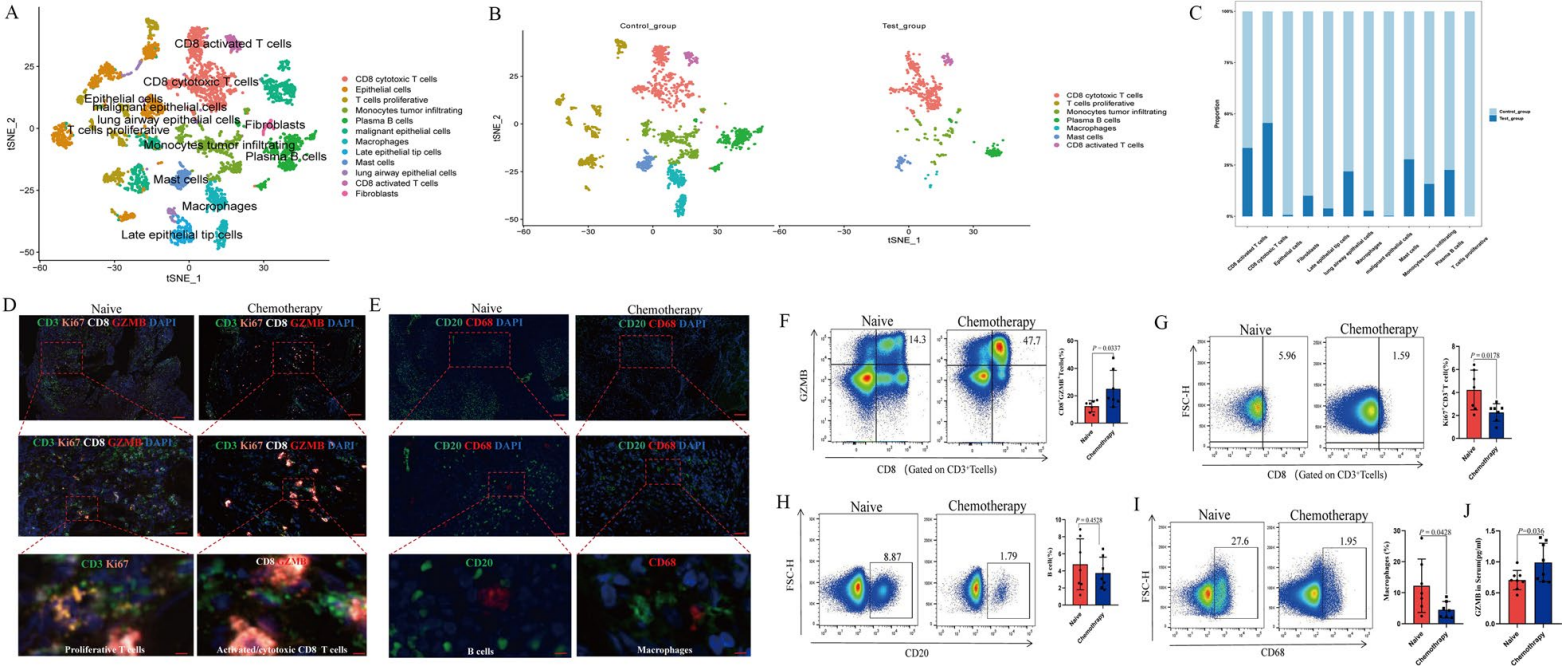
**A** TSNE of different types of immune cells, with each cell color coded for its sample type of origin based on the transcript features. **B** The TSNE plot of immune cells within the tumor microenvironment in patients with non-NAC and NAC treatment. **C** The proportion of immune cells in SCLC patients with non-NAC and NAC treatment. **D** Multiplex immunofluorescence images of T cell infiltration in paired pre-NAC and post-NAC specimens of SCLC. **E** Multiplex immunofluorescence images of B cell and macrophage infiltration in paired pre-NAC and post-NAC specimens of SCLC. **F** Comparison of CD8 + GZMB + T cell infiltration in paired pre-NAC and post-NAC PBMC of SCLC, as detected by flow cytometry. **G** Comparison of Ki67 + CD3 + T cell infiltration in paired pre-NAC and post-NAC PBMC of SCLC, as detected by flow cytometry. **H** Comparison of B cell infiltration in pre-NAC and post-NAC PBMC of SCLC, as detected by flow cytometry. **I** Comparison of macrophage infiltration in PBMC pre-NAC and post-NAC SCLC, as detected by flow cytometry. **J** Comparison of serum GZMB in pre-NAC and post-NAC of SCLC, as detected by ELISA

Additionally, we analyzed and compared the TME in paratumoral tissues between SCLC patients with and without NAC treatment, which was consistent with the milieu in tumoral tissues between non-NAC-treated and NAC-treated SCLC patients (Additional file 2: Fig. S2).

To validate the TME alteration incurred by NAC, we next recruited a total of 8 histologically confirmed SCLC patients from Shandong Cancer Hospital and Institute. Their tumor tissues and peripheral blood were both collected prior to and after six cycles of EP/EC chemotherapy. Their baseline characteristics were demonstrated in Additional file 4: Table S1. Then we performed multiplex immunofluorescence (mIF) staining of a panel of immune markers (CD3, CD8, Ki67 and GZMB) to phenotype and enumerate immune cell subpopulations in these SCLC samples, which revealed the more infiltration of GZMAB + CD8 T cells and less infiltration of T cell proliferative cells in SCLC patients with exposure to chemotherapy (Fig. 1D). Results also found that less amounts of B cells (CD20) and macrophages (CD68) were detected after chemotherapy, as shown in Fig. 1E. Peripheral blood mononuclear cells (PBMCs) were collected from peripheral blood and then stimulated by cell stimulation cocktail, after which were stained with CD8, GAZMB, CD3, CD20 and CD68. The details of antibodies against mIF and flow cytometry were shown in Additional file 5: Table S2 and Additional file 6: Table S3, respectively. The dots plots and gating strategies to determine the lymphocyte and macrophages were shown in Additional file 3: Fig. S3A, B. The proportion of CD8 + GZMB + T cells and TNF-α + CD8 + T cells were significantly increased in the PBMCs from patients with SCLC undergoing chemotherapy, as shown in Fig. 1F and Additional file 3: Fig. S3C (P = 0.0337 and P = 0.0458 respectively). And the ratio of CD8 + γ-IFN + T cells was numerically upregulated in the PBMCs from patients with SCLC undergoing chemotherapy (Additional file 3: Fig. S3D). The ratio of CD3 + Ki67 + T cells and macrophages were significantly decreased after chemotherapeutic treatments in SCLC patients (P = 0.0178 and P = 0.0428 respectively) **(**Fig. 1G, I) and the proportion of B cell was numerically downregulated in SCLC patients after chemotherapy (P = 0.4528) (Fig. 1H). Besides, the amount of serum GZMB was significantly reduced after chemotherapy, as tested by enzyme-linked immunosorbent assay (ELISA) (P = 0.036) (Fig. 1J).

NAC, a therapeutic strategy to achieve curative surgical resection and reduce the possibility of metastasis, has been reported to improve survival and widely used among cancer patients [4]. With the advent of immunotherapy, amounting evidence has demonstrated the immunomodulatory effects of conventional chemotherapy [5]. However, studies are scarce on the influence of NAC on the TME in SCLC. We not only analyzed the NAC-induced TME alteration in SCLC by utilizing single-cell RNA sequencing, but also coupled tissue analyses with peripheral blood to validate TME changes and explore the TME alterations after exposure to chemotherapy in SCLC.

Our study demonstrated NAC increased activated and cytotoxic CD8 + T cell and decreased proliferative T cell, B cell and macrophage in SCLC. Our findings suggest that neoadjuvant chemo-immunotherapy could be a potential treatment option for patients with resectable SCLC. Meanwhile, our team has been conducting a phase 2 clinical trial exploring the role of neoadjuvant chemo-immunotherapy in patients with locally advanced SCLC, which might change the locally advanced SCLC as a potentially lethal malignancy into a curable disease in the clinical setting.

### Supplementary Information

**Additional file 1: Figure S1. A** The correlation between the number of unique molecular identifiers (UMIs) and the number of genes. **B** Principal analyses of the top 2000 variant genes using RunPCA. **C** Visualization of clustering using DimPlot. **D** Stratification and cell-type identification of single cells from 11 SCLC cases. Different types of cells were grouped into distinctive cell clusters. **E.** The infiltration of subpopulation of immune cells in tumor tissues in 2 NAC-treated SCLC patients and 9 non-NAC-treated SCLC patients prior to surgical resection.

**Additional file 2: Figure S2. A** tSNE of different immune cells in para-tumoral tissue with each cell color coded for the associated cell type. **B** tSNE of different immune cells in para-tumoral tissue with each cell color coded for the treatment type. C. The infiltration of subpopulation of immune cells in para-tumoral tissues in 2 NAC-treated SCLC patients and 9 non-NAC-treated SCLC patients prior to surgical resection.

**Additional file 3: Figure S3. A** The dots plots and gating strategies to determine the lymphocyte were shown. **B** The dots plots and gating strategies to determine the macrophages were shown **C** Comparison of TNF-α + CD8 + T cell infiltration in pre-NAC and post-NAC PBMC of SCLC, as detected by flow cytometry. **D** Comparison of γ-IFN + CD8 + T cell infiltration in pre-NAC and post-NAC PBMC of SCLC, as detected by flow cytometry.

**Additional file 4: Table S1**. The baseline characteristics of 8 SCLC patients.

**Additional file 5: Table S2. **Staining used for multiplex immunofluorescence.

**Additional file 6: Table S3. **Antibodies for flow cytometry staining.

**Additional file 7: **Methods.

## Data Availability

Data are available upon reasonable request. The data that support the findings of this study are available from the corresponding author upon request.
