# Supplementary figures and images for: Neoadjuvant chemotherapy remodels the tumor immune microenvironment by increasing activated and cytotoxic T cell, decreasing B cells and macrophages in small cell lung cancer
Source: J Transl Med. 2023 Sep 21;21:645. doi: 10.1186/s12967-023-04526-4 (PMC10512529; doi:10.1186/s12967-023-04526-4)

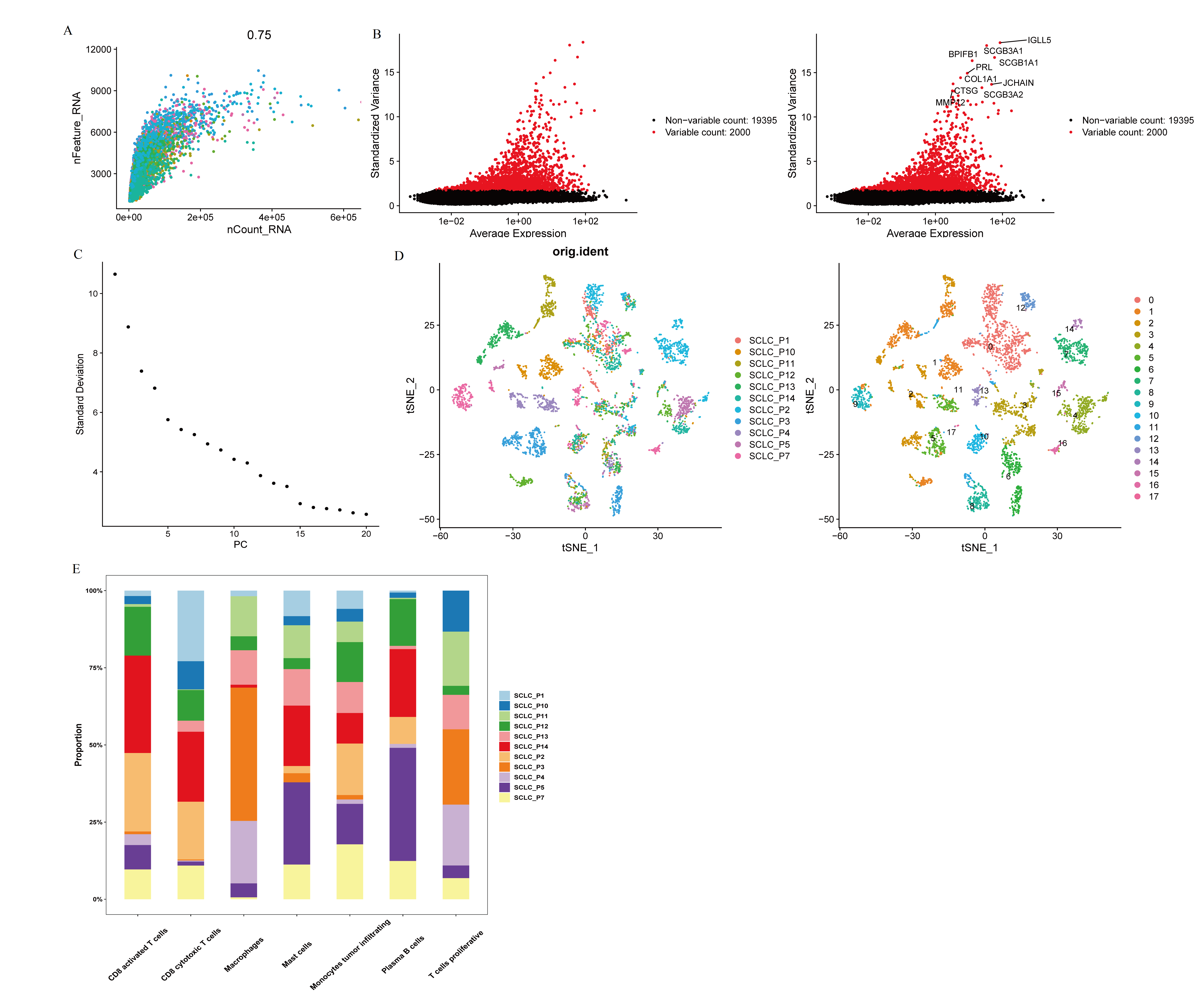

Supplement: Supplementary file 1 — Additional file 1: Figure S1. A The correlation between the number of unique molecular identifiers (UMIs) and the number of genes. B Principal analyses of the top 2000 variant genes using RunPCA. C Visualization of clustering using DimPlot. D Stratification and cell-type identification of single cells from 11 SCLC cases. Different types of cells were grouped into distinctive cell clusters. E. The infiltration of subpopulation of immune cells in tumor tissues in 2 NAC-treated SCLC patients and 9 non-NAC-treated SCLC patients prior to surgical resection. [file 12967_2023_4526_MOESM1_ESM.tif]

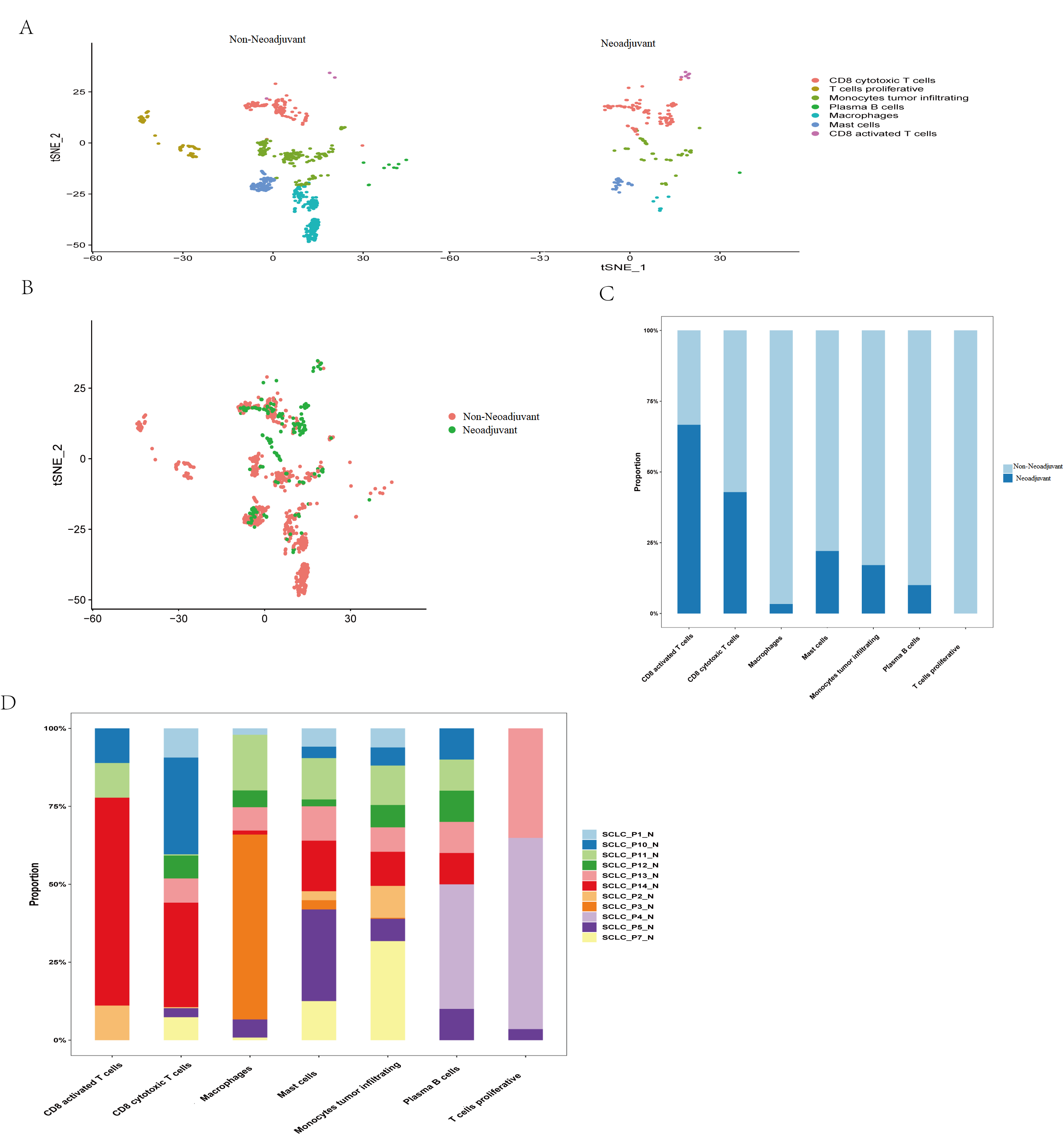

Supplement: Supplementary file 2 — Additional file 2: Figure S2. A tSNE of different immune cells in para-tumoral tissue with each cell color coded for the associated cell type. B tSNE of different immune cells in para-tumoral tissue with each cell color coded for the treatment type. C. The infiltration of subpopulation of immune cells in para-tumoral tissues in 2 NAC-treated SCLC patients and 9 non-NAC-treated SCLC patients prior to surgical resection. [file 12967_2023_4526_MOESM2_ESM.tif]

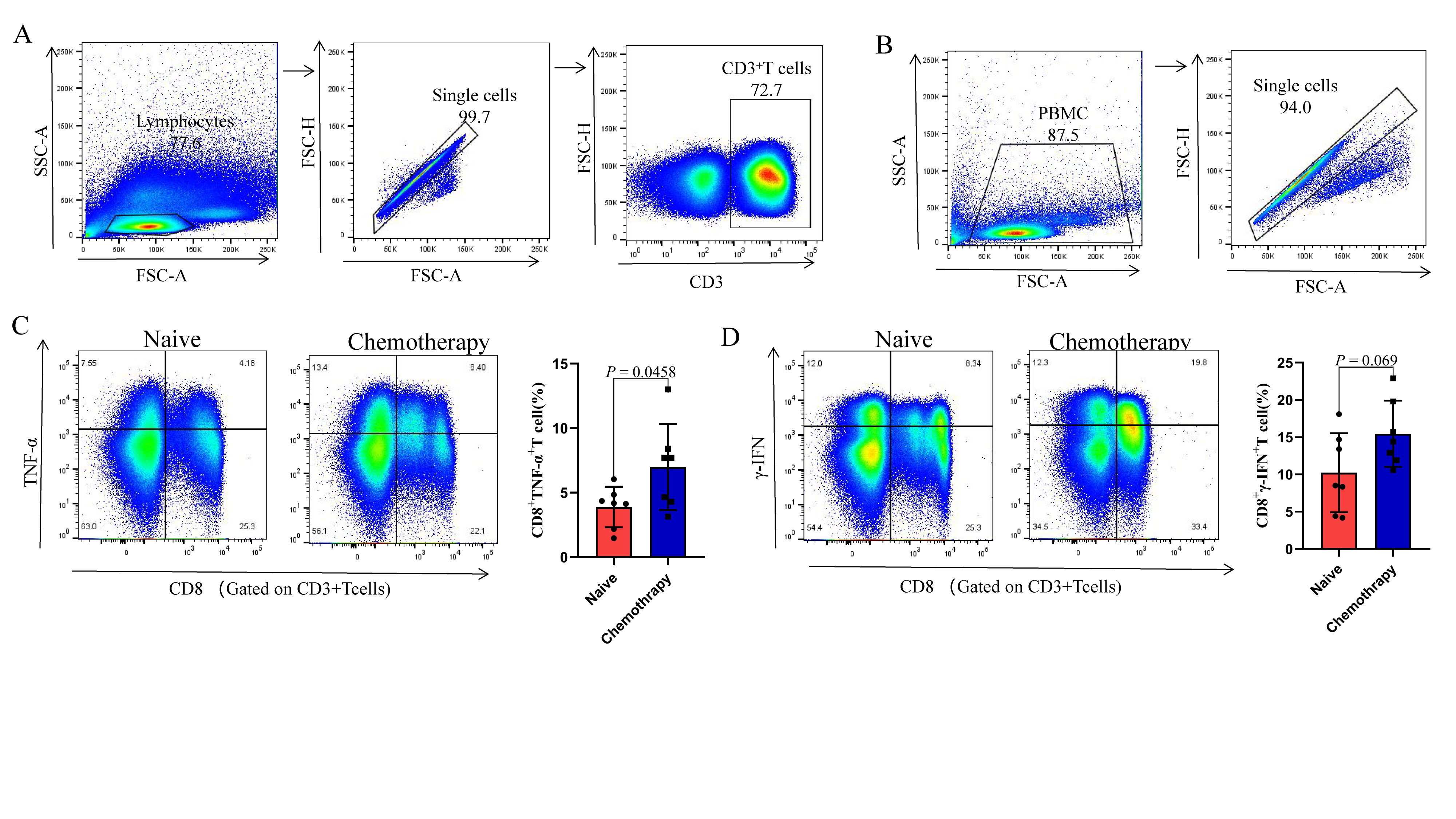

Supplement: Supplementary file 3 — Additional file 3: Figure S3. A The dots plots and gating strategies to determine the lymphocyte were shown. B The dots plots and gating strategies to determine the macrophages were shown C Comparison of TNF-α + CD8 + T cell infiltration in pre-NAC and post-NAC PBMC of SCLC, as detected by flow cytometry. D Comparison of γ-IFN + CD8 + T cell infiltration in pre-NAC and post-NAC PBMC of SCLC, as detected by flow cytometry. [file 12967_2023_4526_MOESM3_ESM.tif]
