# Supplementary material for: Neoadjuvant chemotherapy remodels the tumor immune microenvironment by increasing activated and cytotoxic T cell, decreasing B cells and macrophages in small cell lung cancer
Source: J Transl Med. 2023 Sep 21;21:645. doi: 10.1186/s12967-023-04526-4 (PMC10512529; doi:10.1186/s12967-023-04526-4)
Supplement: Supplementary file 4 — Additional file 4: Table S1. The baseline characteristics of 8 SCLC patients. [file 12967_2023_4526_MOESM4_ESM.docx]

**Table S1. The baseline characteristics of 8 SCLC patients.**

| **Patient ID** | **Histology** | **Smoking history** | **Chemotherapy strategy** | **FFPE** | **PBMC** | **Serum** |
| --- | --- | --- | --- | --- | --- | --- |
| 1 | SCLC | Never | EP | Yes | Yes | Yes |
| 2 | SCLC | Never | EP |  | Yes | Yes |
| 3 | SCLC | Never | EC | Yes | Yes | Yes |
| 4 | SCLC | Never | EP | Yes | Yes | Yes |
| 5 | SCLC | Current | EC | Yes | Yes | Yes |
| 6 | SCLC | Current | EC |  |  | Yes |
| 7 | SCLC | Current | EP | Yes | Yes | Yes |
| 8 | SCLC | Current | EC |  | Yes | Yes |

Abbreviations: EP: etoposide combined with cisplatin; EC: etoposide combined with carboplatin
