# Supplementary material for: Neoadjuvant chemotherapy remodels the tumor immune microenvironment by increasing activated and cytotoxic T cell, decreasing B cells and macrophages in small cell lung cancer
Source: J Transl Med. 2023 Sep 21;21:645. doi: 10.1186/s12967-023-04526-4 (PMC10512529; doi:10.1186/s12967-023-04526-4)
Supplement: Supplementary file 5 — Additional file 5: Table S2. Staining used for multiplex immunofluorescence. [file 12967_2023_4526_MOESM5_ESM.docx]

**Table S2.** Staining used for multiplex immunofluorescence

| **Number** | **Molecules** | **Staining** |
| --- | --- | --- |
|  |  |  |
| 1 | CD20 | Opal 480 |
| 2 | Ki67 | Opal 620 |
| 3 | CD3 | Opal 520 |
| 4 | GZMB | Opal 690 |
| 5 | CD68 | Opal 570 |
| 6 | CD8 | Opal 780 |
| 8 | DAPI | / |
