# Supplementary material for: Neoadjuvant chemotherapy remodels the tumor immune microenvironment by increasing activated and cytotoxic T cell, decreasing B cells and macrophages in small cell lung cancer
Source: J Transl Med. 2023 Sep 21;21:645. doi: 10.1186/s12967-023-04526-4 (PMC10512529; doi:10.1186/s12967-023-04526-4)
Supplement: Supplementary file 6 — Additional file 6: Table S3. Antibodies for flow cytometry staining. [file 12967_2023_4526_MOESM6_ESM.docx]

**Table S3.** Antibodies for flow cytometry staining

| **Number** | **Targeted molecules** | **Staining** | **Catalogue number** |
| --- | --- | --- | --- |
| 1 | Anti-human CD8a | FITC | Biolegend, 301006 |
| 2 | Anti-human Ki67 | Percp/Cyanine 5.5 | Biolegend, 350519 |
| 3 | Anti-human CD3 | PE | Biolegend, 317308 |
| 4 | Anti-human/mouse Granzyme B Recombinant Antibody | APC | Biolegend, 372203 |
| 5 | Anti-human IFN-γ | APC/Cyanine 7 | Biolegend, 506523 |
| 6 | Anti-human TNF-α | Alexa Fluor 700 | Biolegend, 502927 |
| 8 | Anti-human CD68 | PE | Biolegend, 333807 |
| 9 | Anti-human CD20 | FITC | Biolegend, 302303 |
