# Supplementary material for: Neoadjuvant chemotherapy remodels the tumor immune microenvironment by increasing activated and cytotoxic T cell, decreasing B cells and macrophages in small cell lung cancer
Source: J Transl Med. 2023 Sep 21;21:645. doi: 10.1186/s12967-023-04526-4 (PMC10512529; doi:10.1186/s12967-023-04526-4)
Supplement: Supplementary file 7 — Additional file 7: Methods. [file 12967_2023_4526_MOESM7_ESM.docx]

**Additional file 7: methods**

We analyzed the original data by Counts matrix using the Seurat package in R. A total of 5025 cells were obtained. Data on genomic expression were standardized using the function of “NormalizeData”. Errors on UMI and mitochondrial gene expression were corrected using regression analyses by the function of “ScaleData”. Expression matrix after correction were used for cell clustering and dimensionality reduction. As the amounts of sequencing data increases, the amount of both genes that have been detected by single-cell RNA sequencing and UMI also increase. The clusters of cells that deviate from other cells were possibly the multiple cells. Dimensionality reduction was achieved using the Seurat package, before which a total of 2000 genes with high variability (HVGs) was selected in expression matrix after correction. Eigenvalue were then obtained by the function of “FindVariableGenes” in Seurat package. Analyses of principal component in the top 2000 HVGS were performed using the function of “RunPCA”. In order to eliminate signal to noise ratio, the important principal components were chosen by the function of “ElbowPlot” and achieved by permutation test. Cells were clustered in PCA space using “FindCluster” function. Parameter resolution was set at 0.8, which was used to differentiate the main cell types, such as T cell, B cell or macrophage. Next, cell clusters were projected into two-dimensional space using “RunMAP”. The visualization of clustering was achieved using “DimPlot” function.
